# Monte Carlo simulated data for multi-criteria selection of city and compact electric vehicles in Poland

**DOI:** 10.1016/j.dib.2021.107118

**Published:** 2021-05-08

**Authors:** Paweł Ziemba

**Affiliations:** Faculty of Economics, Finance and Management, University of Szczecin, Cukrowa 8, Szczecin 71-004, Poland

**Keywords:** Sustainable transportation, Electric vehicles, Multi-criteria decision aid, NEAT F-PROMETHEE, Robustness analysis, Fuzzy sets, Stochastic analysis, Uncertainty

## Abstract

The data presented in this article describes a multi-criteria decision problem, where 13 criteria and 14 alternatives have been taken into account, consisting in the selection of an electric vehicle. The data set contains: (1) the parameters of the electric vehicles concerned included in the alternative performance model, (2) the weights of the criteria for assessing the vehicles, preference functions and thresholds constituting the preference model, (3) the overall performances and rankings of the alternatives (electric vehicles concerned). The data on vehicle parameters were collected from reports, catalogues and websites of car manufacturers and then processed into a decision table. In turn, data constituting various random preference models were generated using the Monte Carlo method. The overall performances and ranks of the alternatives were obtained using the MCDA (multi-criteria decision aid) method called NEAT F-PROMETHEE (New Easy Approach To Fuzzy Preference Ranking Organization METHod for Enrichment Evaluation), based on the performance model (decision table) and individual preference models. By linking vehicle parameters, preference models and vehicle rankings, the data allow, among other things, determining the impact of the preference model (weights of criteria, preference functions, thresholds) on the obtained vehicle rankings. The data also allow determining the probability of individual vehicles taking a specific position in the ranking on the basis of vehicle parameters, and regardless of the preferences of decision makers. Therefore, the data presented are valuable for practitioners and theorists dealing with electric vehicles and management, and in particular decision support. In the context of decision support, this data is also valuable to consumers considering the purchase of an electric vehicle, electric vehicle manufacturers, and dealers because it indicates the vehicles with the greatest market potential and user acceptance. This fact was confirmed by the research article entitled “Multi-criteria approach to stochastic and fuzzy uncertainty in the selection of electric vehicles with high social acceptance” [1] linked to this data article.

## Specifications Table

**Table:** 

Subject	Management Science and Operations Research
Specific subject area	Multi-criteria Decision Aid, robustness analysis, sustainable transportation, electric vehicles
Type of data	Tables Figures
How data were acquired	Input data concerning parameters of electric vehicles were collected from manufacturers' websites, reports and catalogues of electric vehicles. Input data for other parameters of the decision problem were generated using a Monte Carlo simulation. Output data were generated using the MCDA (multi-criteria decision aid) method called NEAT F-PROMETHEE (New Easy Approach To Fuzzy Preference Ranking Organization METHod for Enrichment Evaluation), based on the input data.
Data format	Raw Processed Simulated
Parameters for data collection	Input data on the parameters of electric vehicles were collected for city and compact cars, at least 4-seater ones, available for sale on the Polish market in 2021. Other input data and output data were generated taking into account the principles resulting from the application of the NEAT F-PROMETHEE method.
Description of data collection	The input data concerning the parameters of electric vehicles were obtained from [2,3] and websites of vehicle manufacturers. Input data concerning preference models (weights of criteria, preference functions, preference thresholds) were generated using the Monte Carlo method. The processing of input data and generation of output data (overall performances of alternatives, rankings of alternatives) was carried out using the NEAT F-PROMETHEE method. The data were saved in the XLSX and CSV data files.
Data source location	Institution: Polish Alternative Fuels Association City/Town/Region: Warszawa Country: Poland https://pspa.com.pl/media/2020/08/katalog_pojazdow_elektrycznych_2020_S_.pdf Institution: Electric Vehicle Database https://ev-database.org/car/1164/Renault-Zoe-ZE50-R110 https://ev-database.org/car/1205/Renault-Zoe-ZE50-R135 https://ev-database.org/car/1232/Smart-EQ-forfour https://ev-database.org/car/1145/BMW-i3–120-Ah https://ev-database.org/car/1149/BMW-i3s-120-Ah https://ev-database.org/car/1409/Mini-Cooper-SE
	https://ev-database.org/car/1192/Opel-Corsa-e https://ev-database.org/car/1168/Peugeot-e-208 https://ev-database.org/car/1127/Volkswagen-ID3-Pure-Performance https://ev-database.org/car/1202/Volkswagen-ID3-Pro https://ev-database.org/car/1203/Volkswagen-ID3-Pro-S https://ev-database.org/car/1165/Hyundai-IONIQ-Electric https://ev-database.org/car/1106/Nissan-Leaf https://ev-database.org/car/1144/Nissan-Leaf-eplus
Data accessibility	Repository name: Mendeley Data Direct URL to data: http://dx.doi.org/10.17632/c9ft5p6yby.1
Related research article	Paweł Ziemba, Multi-criteria approach to stochastic and fuzzy uncertainty in the selection of electric vehicles with high social acceptance, Expert Systems with Applications 173 (2021) 114,686, https://doi.org/10.1016/j.eswa.2021.114686[1]

## Value of the Data

• The data set contains parameters of city and compact electric vehicles available on the Polish market. The data set also contains simulation data for selection of specific cars based on their parameters and specific preferences of the decision-maker. The data set allows for a thorough analysis of how the decision-maker's preferences affect the selection of a specific car as the best decision-making alternative.
• The data set contains, among others, rankings of electric vehicles established on the basis of many random preference models of the decision-maker. Therefore, the data allow determining with great accuracy the probability of individual vehicles taking a specific position in the ranking. The rank is predicted on the basis of vehicle parameters, and regardless of the preferences of the decision-makers. In other words, the data allow us to determine the chance of a vehicle being selected by a consumer looking to purchase an electric vehicle. Therefore, the data are useful for vehicle manufacturers and sellers, as well as for consumers considering the purchase of an electric vehicle.
• By linking vehicle parameters, preference models and vehicle rankings, the data allow determining how to influence the decision-maker (in which direction to change the decision-maker's preferences) to purchase the indicated vehicle. As a result, the data may indicate the directions of marketing activities.
• The output data (rankings and overall performances of the alternatives) were generated using the NEAT F-PROMETHEE method on the basis of the input data. Therefore, the data set is helpful in analyzing the functioning and understanding of this relatively new decision aid method.
• Input data on the parameters of electric vehicles were collected on the basis of the analysis of the literature (vehicle manufacturers' websites, reports, catalogues). This will allow other researchers to use this data, shortening their search time.

## 1 Data Description

**Fig. 1:**
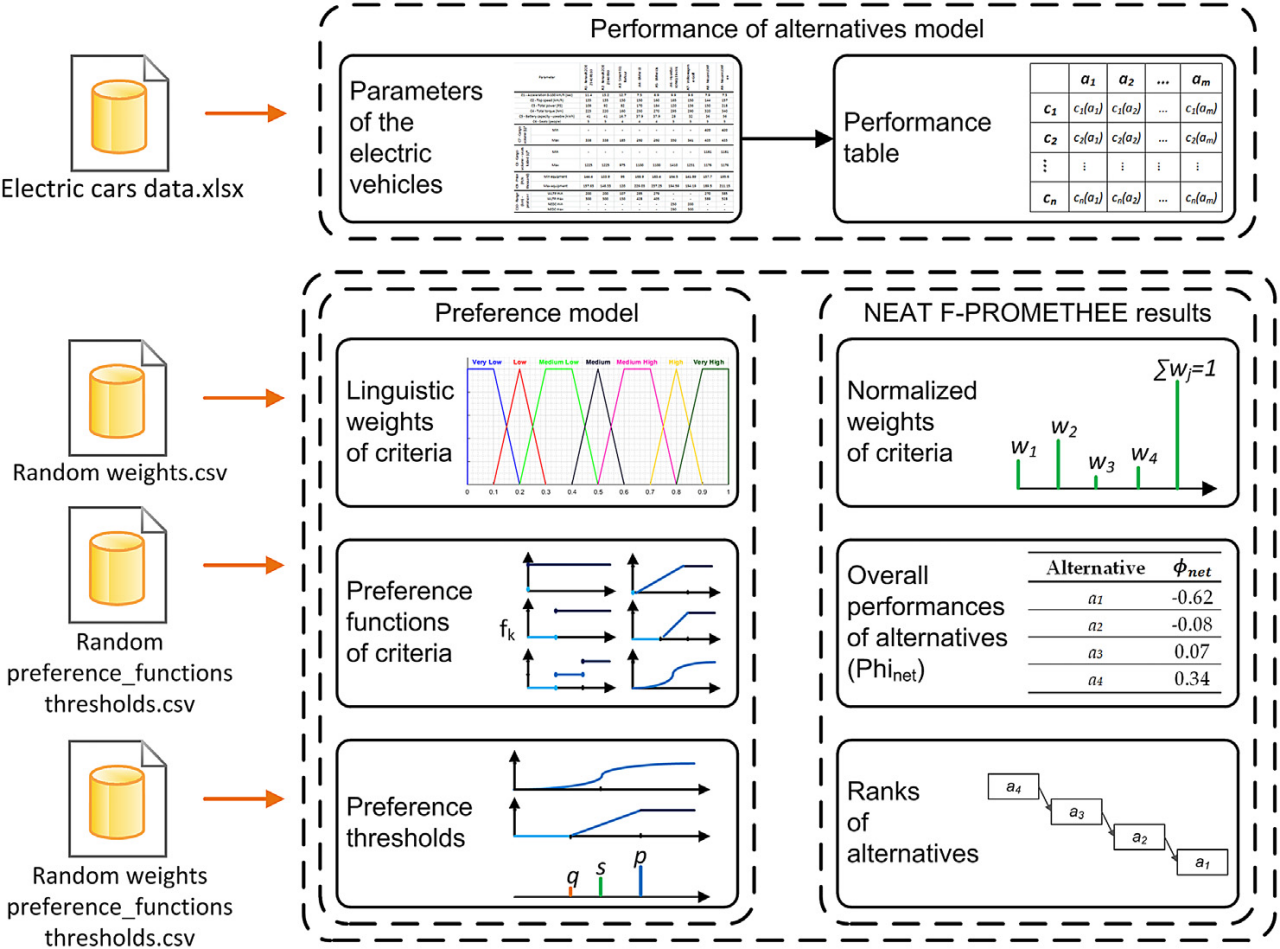
Data model and their arrangement in data files.

This article presents data related to the management decision problem of evaluating electric vehicles using the MCDA (multi-criteria decision aid) method called NEAT F-PROMETHEE (New Easy Approach To Fuzzy Preference Ranking Organization METHod for Enrichment Evaluation) [4]. In the data set, data characterizing the decision problem (input data) and its solution (output data) were distinguished. The input data primarily describe the performance model of decision alternatives in the form of objective quantitative criteria, both certain and uncertain/imprecise, describing the decision alternatives. These data were collected in a raw form and then processed into a performance table with crisp numbers, interval numbers (INs) and trapezoidal fuzzy numbers (TFNs). These are contained in a data file called `Electric cars data.xlsx'. It should be explained here that the decision-making alternatives are the electric vehicles available on the Polish market and, in particular, urban and compact cars (market segment A–C) with at least four seats, as such vehicles are the most popular among consumers. In addition, the input data include linguistic weights of criteria, preference functions of criteria and preference thresholds used in the NEAT F-PROMETHEE method and forming a preference model. In turn, the output data show normalized weights of criteria, overall performances of alternatives in the form of the values $\phi_{net}$ and rankings of alternatives. The indicated input and output data are contained in the data files ‘Random weights.csv’, ‘Random preference_functions thresholds.csv’, ‘Random weights preference_functions thresholds.csv’. The data model for the decision problem is shown in Fig. 1.

**Fig. 2:**
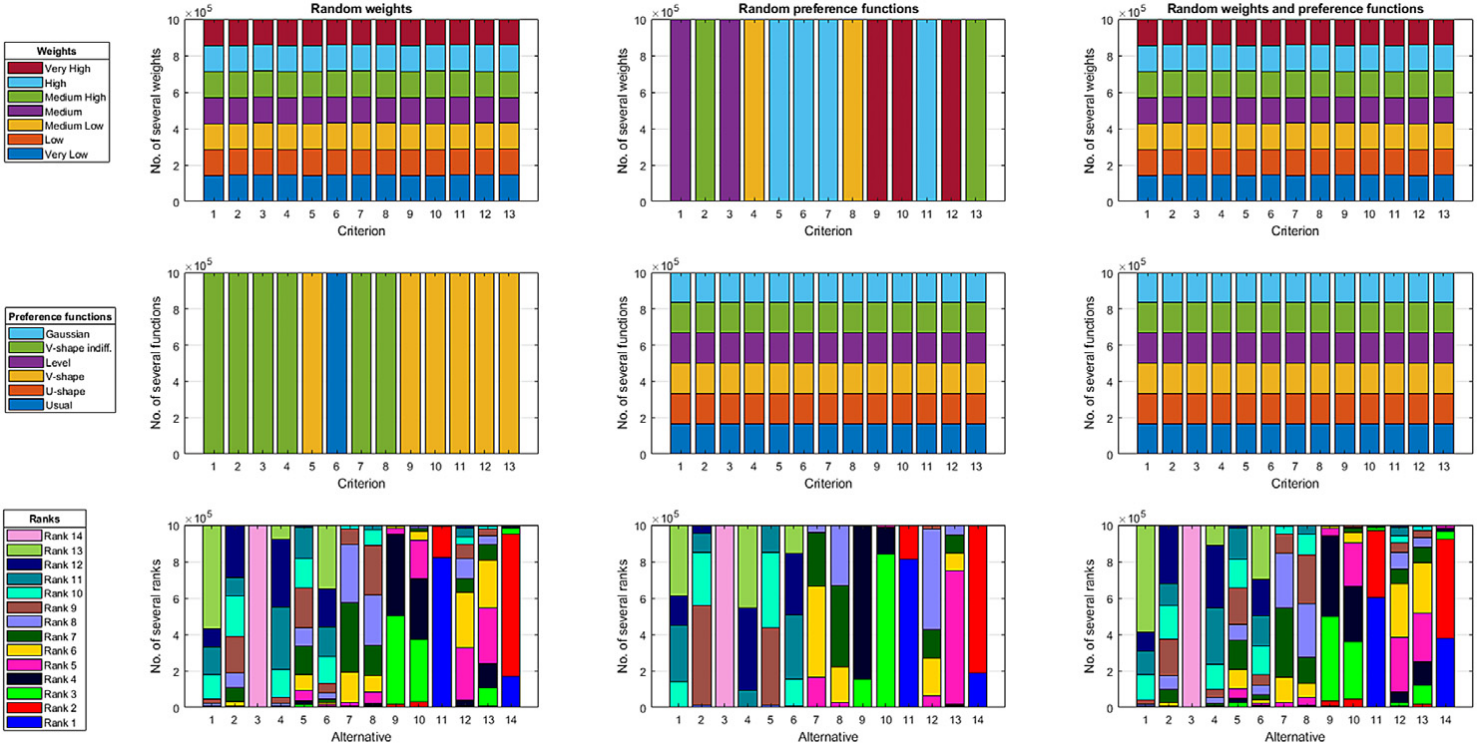
Distribution of weights of criteria, preference function of criteria and ranks of alternatives in the data sets.

Each of these three files contains different sets of input data from the Monte Carlo simulation [5] and their respective output data. The file name describes which input data were generated using the Monte Carlo method. In the file ‘Random weights.csv’, the Monte Carlo method was used to generate the criteria weights (the preference functions and thresholds were fixed). In the file ‘Random preference_functions thresholds.csv’, the preference functions and thresholds were randomly selected (the criteria weights were fixed). In the file ‘Random weights preference_functions thresholds.csv’, the Monte Carlo method was used to generate each of these three decision problem parameters. Each of these three files contains 1 million rows of data, and each row contains data related to one Monte Carlo simulation. The characteristics of the data relating to the linguistic weights of criteria, preference functions and alternative rankings in the files ‘Random weights.csv’, ‘Random preference_functions thresholds.csv’ and ‘Random weights preference_functions thresholds.csv’ are shown in Fig. 2.

The graphs in the rows in Fig. 2 refer, respectively, to the characteristics of the criteria weights, preference functions and alternative rankings. In turn, the columns specify which data were random and which were constant. The first column shows the characteristics of the data file `Random weights.csv', which contains random weights of criteria and constant functions of preferences. The second column describes the data contained in the file ‘Random preference_functions thresholds.csv’ where the weights of the criteria were constant and the preference functions and thresholds were random. The third column shows the characteristics of the data contained in the file ‘Random weights preference_functions thresholds.csv’, which includes both random weights of criteria as well as random preference functions and preference thresholds. Depending on the weights of criteria, preference functions and preference thresholds, the ranks of alternatives were obtained, the characteristics of which are presented in the diagrams in the third row of Fig. 2.

Tables 1–2 provide a formal description of the data files.

**Table 1:** Description of the file ‘Electric cars data.xlsx’.

File name	Electric cars data.xlsx
Description	Performance data of electric vehicles; Performance table of electric vehicles
No of records	14; 14 Records - electric vehicles: • A1 – Renault ZOE R110, • A2 – Renault ZOE R135, • A3 – Smart EQ forfour, • A4 – BMW i3, • A5 – BMW i3s, • A6 – Mini Cooper SE, • A7 – Opel Corsa-e, • A8 – Peugeot e-208, • A9 – Volkswagen ID.3 Pure Performance, • A10 – Volkswagen ID.3 Pro, • A11 – Volkswagen ID.3 Pro-S, • A12 – Hyundai IONIQ Electric, • A13 – Nissan LEAF, • A14 – Nissan LEAF *e*+.
Structure of record	Raw data section – data on the parameters of electric vehicles: • Acceleration 0–100 km/h (*sec*), • Top speed (km/h), • Total power (PS), • Total torque (Nm), • Battery capacity – useable (kWh), • Seats (people), • Cargo volume (L), • Cargo volume– seats folded (L), • Price (PLN thousand) – depending on the equipment version, • Range (km) – depending on weather conditions, road conditions and measurement method, • Charging time (m) – depending on the type of charger used, • Fast charging time (m) – depending on the type of charger used, • Energy consumption (kWh/km) – depending on weather conditions, road conditions and measurement method. Performance table - criteria: • C1 – Acceleration (crisp number represented as a trapezoidal fuzzy number), • C2 – Top speed (crisp number represented as a trapezoidal fuzzy number), • C3 – Total power (crisp number represented as a trapezoidal fuzzy number), • C4 – Total torque (crisp number represented as a trapezoidal fuzzy number), • C5 – Battery capacity – useable (crisp number represented as a trapezoidal fuzzy number), • C6 – Seats (crisp number represented as a trapezoidal fuzzy number), • C7 – Cargo volume (crisp number represented as a trapezoidal fuzzy number), • C8 – Cargo volume – seats folded (crisp number represented as a trapezoidal fuzzy number), • C9 – Price (interval number represented as a trapezoidal fuzzy number), • C10 – Range (trapezoidal fuzzy number), • C11 – Charging time (trapezoidal fuzzy number), • C12 – Fast charging time (trapezoidal fuzzy number), • C13 – Energy consumption (trapezoidal fuzzy number).

**Table 2:** Description of the files ‘Random weights.csv’, ‘Random preference_functions thresholds.csv’ and ‘Random weights preference_functions thresholds.csv’.

File name	Random weights.csv; Random preference_functions thresholds.csv; Random weights preference_functions thresholds.csv
Description	Data on the decision-making problem model
No of records	1 million
Structure of record	Data for thirteen criteria (C1-C13): • Linguistic weights of criteria [1-Very Low; 2-Low; 3-Medium Low; 4-Medium; 5-Medium High; 6-High; 7-Very High] – input data, • Normalized weights of criteria [0:1] – output data, • Preference functions of criteria [1-Usual; 2-U-shape; 3-V-shape; 4-Level; 5-V-shape with indifference area; 6-Gaussian] – input data, • Preference thresholds ○ Indifference thresholds (q) [0:0.75σ]* – input data, ○ Preference thresholds (p) [0.75σ:2.5σ]* – input data, ○ Gaussian thresholds (s) [σ]* – input data. Data for fourteen alternatives (A1-A14): • Overall performances of alternatives (Phi_net_ values) [−1:1] – output data, • Ranks of alternatives [1:14] – output data.

⁎ The value σ was calculated for each criterion separately as a population standard deviation, where the population was the value of all alternatives. In the case of interval numbers and trapezoidal fuzzy numbers, each point determining the number was taken into account.

## 2 Experimental Design, Materials and Methods

As part of the research experiment, data describing the performances of decision-making alternatives were collected and processed in the form of a performance table. In addition, constant values describing the decision maker's preferences were expertly defined and then new values of the decision maker's preferences were randomly generated using the Monte Carlo method. Based on these values, data describing the overall performances of the alternatives and their ranks using the NEAT F-PROMETHEE method were obtained.

The performance model of the alternatives was constructed based on the data describing the parameters of electric vehicles, collected from the Polish Alternative Fuels Association [2], Electric Vehicle Database [3], and vehicle manufacturers websites. The collected data concerned electric city and compact cars: Renault ZOE R110, Renault ZOE R135, Smart EQ forfour, BMW i3, BMW i3s, Mini Cooper SE, Opel Corsa-e, Peugeot e-208, Volkswagen ID.3 Pure Performance, Volkswagen ID.3 Pro, Volkswagen ID.3 Pro-S, Hyundai IONIQ Electric, Nissan LEAF, Nissan LEAF *e*+. These data were grouped according to categories describing different vehicle parameters (see Table 1) and processed into a performance table. The vehicle parameters were described by single crisp numbers (*x*), ranges (*x_min_:x_max_*) or multiple values (*x_1_, x_2_, …, x_y_*). In order to apply the NEAT F-PROMETHEE method, all data describing the performances of the alternatives were transformed into trapezoidal fuzzy numbers (TFNs) $\tilde{FN}=\left(FN_{1},FN_{2},FN_{3},FN_{4}\right)$. For single crisp numbers, this was done using the formula (1), the conversion of ranges to TFNs is shown in the formula (2), and the aggregation of multiple values to a TFN is described in the formula (3).

(1) $$FN_{1}=x;\;FN_{2}=x;\;FN_{3}=x;\;FN_{4}=x$$

(2) $$FN_{1}=x_{min};\;FN_{2}=x_{min};\;FN_{3}=x_{max};\;FN_{4}=x_{max}$$

(3) $$FN_{1}=\min_{i=1\ldots y}x_{i};\;FN_{2}=FN_{1}+\frac{\left(\bar{x}-FN_{1}\right)}{2};\;FN_{3}=\bar{x}+\frac{\left(FN_{4}-\bar{x}\right)}{2};\;FN_{4}=\max_{i=1\ldots y}x_{i}$$

The preference model was expertly defined and is presented in Table 3.

**Table 3:** Initial preference model for the decision-making problem of electric vehicle selection.

				Preference thresholds	
Criterion	Preference direction	Weight	Preference function	Indifference threshold (*q*)	Preference threshold (*p*)
C1 - Acceleration	Min	4	5	1.14	2.28
C2 - Top speed	Max	5	5	5.76	11.53
C3 - Total power	Max	4	5	21.12	42.25
C4 - Total torque	Max	3	5	26.38	52.75
C5 - Battery capacity	Max	6	3	–	10.05
C6 - Seats	Max	6	1	–	–
C7 - Cargo volume	Max	6	5	37.41	74.83
C8 - Cargo volume – seats folded	Max	3	5	56.45	112.90
C9 – Price	Min	7	3	–	34.49
C10 – Range	Max	7	3	–	110.3
C11 – Charging time	Min	6	3	–	460.49
C12 – Fast charging time	Min	7	3	–	26.49
C13 – Energy consumption	Min	5	3	–	5.15

Weight: 1-Very Low; 2-Low; 3-Medium Low; 4-Medium; 5-Medium High; 6-High; 7-Very High; Preference function: 1-Usual, 3-V-shape, 4-Level, 5-V-shape with indifference area.

**Fig. 3:**
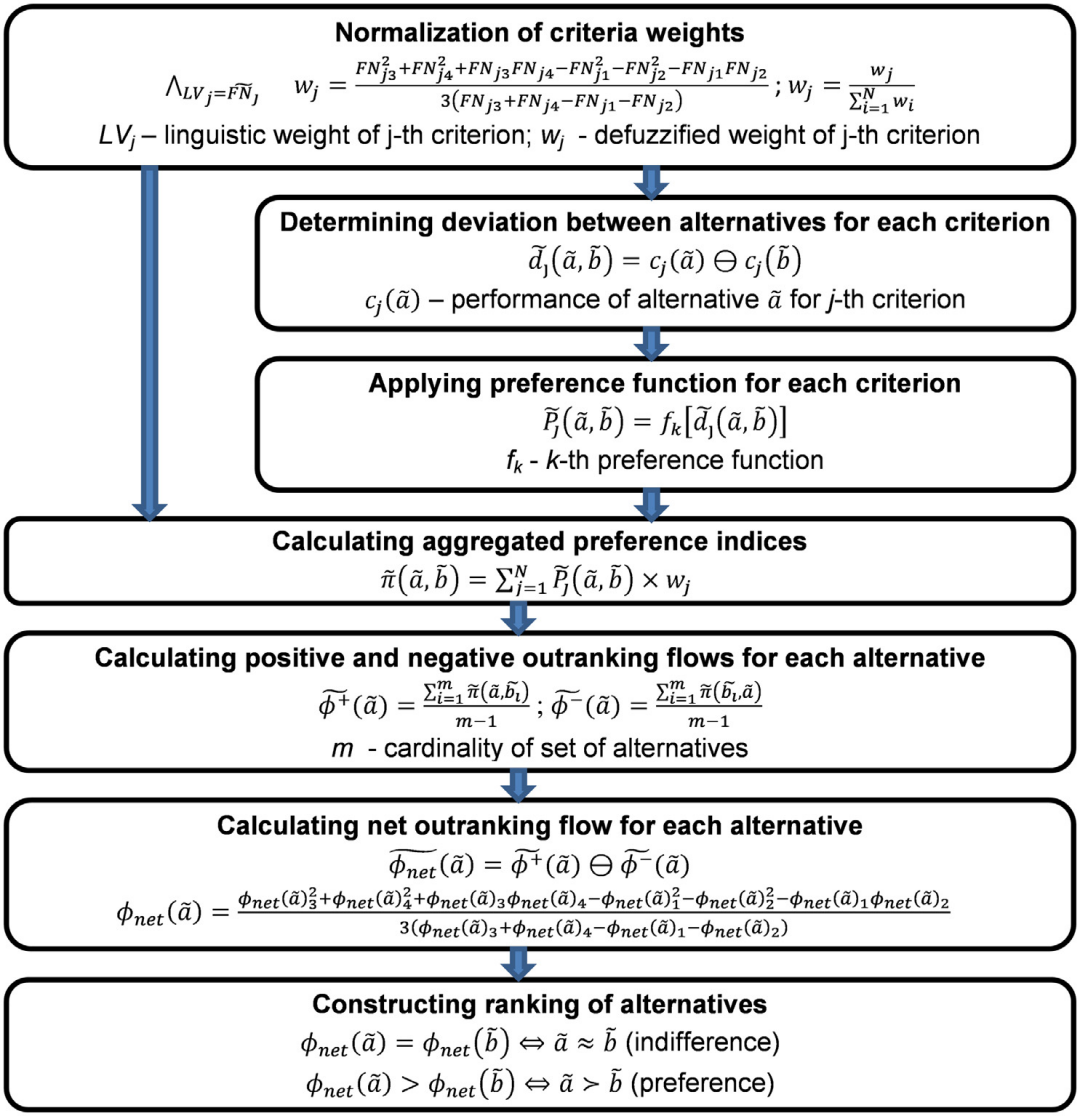
Calculation procedure of NEAT F-PROMETHEE.

Then the individual elements of the preference model (linguistic weights of criteria, preference functions, preference thresholds) were replaced by random values generated based on the Monte Carlo method. The random values were generated using a uniform distribution. The approach used to generate the random values is described by the formula (4).

(4) $$\wedge_{i=1\ldots M}\wedge_{j=1\ldots N}r_{j}^{i}\;\begin{array}{c} i.i.d.\\ ∼ \end{array}\;U\left(v\right)$$

where: $r_{j}^{i}$ denotes a *j*-th random variable generated in an *i* th Monte Carlo iteration for parameters *v* of the uniform distribution *U, i.i.d.* denotes independent and identically distributed. For both the linguistic weights of the criteria, as well as for the preference functions and thresholds *q* and *p*, the number of values generated in each iteration corresponded to the number of criteria (*N* = *13*). The number of iterations was *M* = *1 million* which gives a precision of 0.001 for 95% confidence [6]. For the linguistic weights of the criteria, a discrete distribution was applied by drawing total values from 1 (Very Low) to 7 (Very High) (*v={1,2,…,7}*). For the preference function a discrete distribution was also applied by drawing integers from the range from 1 (Usual criterion) to 6 (Gaussian criterion) (*v={1,2,…,6}*). For thresholds *q* and *p*, a continuous distribution was used, drawing values from the following ranges: for *q v=(0, 0.75 σ_j_)*, for *p v=(0.75 σ_j_, 2.5 σ_j_)*, where *j* denotes a *j*-th criterion. The performance of alternatives and preference model was transformed into output values using the NEAT F-PROMETHEE method, whose calculation procedure is shown in Fig. 3.

**Fig. 4:**
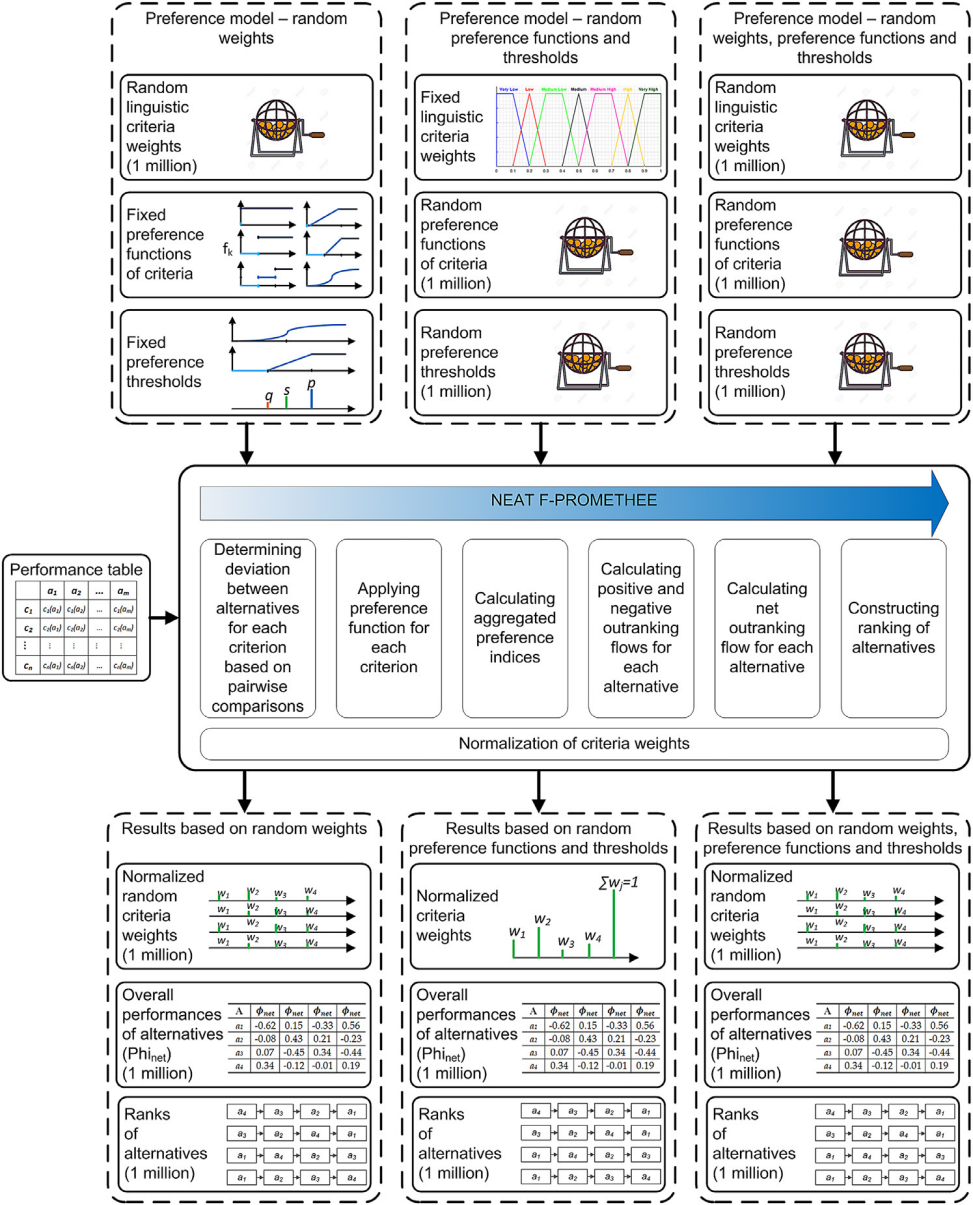
Stochastic analysis framework used to generate data.

The result is three sets of results in the form of normalized weights of criteria, the overall performances of the alternatives *Phi_net_* and ranks of alternatives. The stochastic analysis framework from which all data were generated is shown in Fig. 4.

The data included in this article, generated with the use of the presented framework, provide important information about the decision problem of choosing an electric vehicle. As a result, the data allow for a broad analysis of both the problem itself and its solution. They allow examining, among other things, the dependence of the positions occupied by the alternatives in the rankings on the weights of particular criteria, or on the applied preference functions. Therefore, the presented data are valuable for practitioners and theorists dealing with MCDA methods. In the context of decision support, this data is also valuable to consumers considering the purchase of an electric vehicle, electric vehicle manufacturers, and dealers because it indicates the vehicles with the greatest market potential and user acceptance. On the other hand, the developed framework of stochastic analysis can be useful for generating data describing other decision-making problems, thus allowing for their broad analysis.

## Ethics Statement

The authors declare that they have no known ethical conflict occurs for the raw data collected, e.g. data collected from websites and companies, reported in this article.

## CRediT Author Statement

**Paweł Ziemba:** Conceptualization, Methodology, Software, Data curation, Validation, Writing - original draft, Funding acquisition.

## Declaration of Competing Interest

The authors declare that they have no known competing financial interests or personal relationships which have or could be perceived to have influenced the work reported in this article.

## Appendix Supplementary materials

Image, application 1

Image, application 2

Image, application 3

Image, application 4

Image, application 5

Image, application 6

Image, application 7
